# Anti-apoptotic and Matrix Remodeling Actions of a Small Molecule Agonist of the Human Relaxin Receptor, ML290 in Mice With Unilateral Ureteral Obstruction

**DOI:** 10.3389/fphys.2021.650769

**Published:** 2021-07-07

**Authors:** Hooi Hooi Ng, Mariluz Soula, Bryan Rivas, Kenneth J. Wilson, Juan J. Marugan, Alexander I. Agoulnik

**Affiliations:** ^1^Department of Human and Molecular Genetics, Herbert Wertheim College of Medicine, Florida International University, Miami, FL, United States; ^2^NIH Chemical Genomics Center, National Center for Advancing Translational Sciences, National Institutes of Health, Bethesda, MD, United States

**Keywords:** relaxin, relaxin family peptide receptor 1, apoptosis, extracellular matrix, unilateral ureteral obstruction

## Abstract

Diseases, such as diabetes and hypertension, often lead to chronic kidney failure. The peptide hormone relaxin has been shown to have therapeutic effects in various organs. In the present study, we tested the hypothesis that ML290, a small molecule agonist of the human relaxin receptor (RXFP1), is able to target the kidney to remodel the extracellular matrix and reduce apoptosis induced by unilateral ureteral obstruction (UUO). UUO was performed on the left kidney of humanized RXFP1 mice, where the right kidneys served as contralateral controls. Mice were randomly allocated to receive either vehicle or ML290 (30 mg/kg) *via* daily intraperitoneal injection, and kidneys were collected for apoptosis, RNA, and protein analyses. UUO significantly increased expression of pro-apoptotic markers in both vehicle- and ML290-treated mice when compared to their contralateral control kidneys. Specifically, *Bax* expression and Erk1/2 activity were upregulated, accompanied by an increase of TUNEL-positive cells in the UUO kidneys. Additionally, UUO induced marked increase in myofibroblast differentiation and aberrant remodeling on the extracellular matrix. ML290 suppressed these processes by promoting a reduction of pro-apoptotic, fibroblastic, and inflammatory markers in the UUO kidneys. Finally, the potent effects of ML290 to remodel the extracellular matrix were demonstrated by its ability to reduce collagen gene expression in the UUO kidneys. Our data indicate that daily administration of ML290 has renal protective effects in the UUO mouse model, specifically through its anti-apoptotic and extracellular matrix remodeling properties.

## Introduction

Chronic kidney failure is characterized by a progressive decline in kidney function, accompanied by a decrease in glomerular filtration rate. Primary causes of chronic kidney failure include diabetes and hypertension. The peptide hormone relaxin has potent anti-fibrotic and remodeling properties in various organs (Ng et al., 2019b), demonstrates clinical safety (Dschietzig et al., 2009; Dahlke et al., 2015), and may provide novel therapeutics for chronic kidney failure.

Long-term relaxin treatment modulates the renal hemodynamic changes to increase cardiac output and global arterial compliance, decrease systemic vascular resistance, with maintenance of the mean arterial pressure in conscious male and female normotensive and hypertensive rats (Debrah et al., 2005a). Similar hemodynamic changes are also observed in the angiotensin II-induced hypertensive model, but not in the spontaneously hypertensive and normotensive rats (Debrah et al., 2005b). Moreover, chronic infusion of relaxin in hypertensive rats attenuates renal inflammation, collagen, and lipid accumulation to improve overall renal function (Wang et al., 2017). The renoprotective effects of relaxin under hypertensive conditions are well reported in the literature, but the beneficial action of relaxin in the context of diabetes remains controversial. For example, relaxin fails to protect the kidneys from diabetes-mediated injuries in models of streptozotocin-induced endothelial nitric oxide synthase (NOS) knockout mice (Dschietzig et al., 2015) or the diabetic transgenic mRen-2 rats (Wong et al., 2013). In the Dahl salt-sensitive/resistant rats administered 8% NaCl diet, relaxin treatment reduces blood pressure, TGF-β level, and αSMA expression and increases neuronal NOS and endothelial NOS expression (Yoshida et al., 2012). Apart from the actions of relaxin treatment under diseased conditions, relaxin shows renoprotective effects in physiological aging as well. For instance, endogenous relaxin protects the male mice from developing kidney injury, as evidenced by a downregulation of creatinine and proteinuria, as well as a reduction in collagen deposition in the kidneys of wild-type mice when compared to relaxin-deficient mice (Samuel et al., 2004). Additionally, relaxin enhances glomerular filtration rate and effective renal plasma flow, while reducing effective renal vascular resistance and collagen deposition in the Munich-Wistar rats (Danielson et al., 2006). Recent clinical data demonstrate that relaxin increases creatinine clearance and reduces serum creatinine in acute (Teerlink et al., 2013) and chronic (Dschietzig et al., 2009) heart failure settings, respectively.

In the renal circulation, long-term relaxin treatment reduces renal vascular resistance and myogenic reactivity of small renal arteries and increases renal blood flow and glomerular filtration rate in conscious male and female intact or ovariectomized rats (Conrad, 1984). Notably, exogenous relaxin treatment in non-pregnant female and male rats recapitulates the hemodynamic profile associated with pregnancy (Danielson and Conrad, 2003; Danielson et al., 2006). These studies collectively show the important role of relaxin to maintain the renal hemodynamics during pregnancy. The endogenous role of relaxin is well-documented in the ovary (Anand-Ivell and Ivell, 2014), and indeed, women who had pregnancies occur through *in vitro* fertilization, egg donation, and embryo transfer due to ovarian insufficiency have impaired glomerular filtration rate, which would otherwise increase in normal pregnancy (Smith et al., 2006). This is due to the deficiency of ovarian function and corpus luteum in these women that leads to impaired relaxin production (Sherwood, 1994), indicating the vital role of relaxin to mediate gestational hyperfiltration.

Although relaxin has been shown to demonstrate beneficial actions in various models of kidney disease, it is worth noting that relaxin possesses the universal drawback of peptide-based therapies, such as its short half-life *in vivo* (Chen et al., 1993), which requires continuous infusion to achieve sustained dosage for the treatment of chronic diseases. Therefore, our discovery of a non-peptide small molecule agonist of the relaxin family peptide receptor 1 (RXFP1), ML290, as a result of a high-throughput screening and medicinal chemistry optimization campaign (Chen et al., 2013; Xiao et al., 2013) might serve as an alternative to the recombinant peptide ligands. ML290 is a specific agonist for human RXFP1 and does not activate the rodent receptor (Huang et al., 2015). In this study, we utilized a unique strain of mice generated in our laboratory with knock-in of the human RXFP1 (Kaftanovskaya et al., 2019) to test the therapeutic efficacies of ML290 *in vivo*. A plethora of studies reported the anti-fibrotic role of relaxin (Samuel et al., 2016), and we recently showed that ML290 replicates the protective functions of relaxin in a mouse model of liver fibrosis (Kaftanovskaya et al., 2019). On the other hand, the anti-apoptotic role of relaxin is inconsistent, especially in *in vivo* studies, and no studies to date have investigated the effects of ML290 in the context of apoptosis. Earlier work reported that relaxin treatment has no effect on apoptosis in renal fibroblasts (Heeg et al., 2005) or in an experimental model of renal fibrosis (Hewitson et al., 2010). Contrary to these studies, a recent study shows that relaxin attenuates apoptosis in several rodent models of kidney disease, including ischemia-reperfusion injury (Yoshida et al., 2013), aristolochic acid nephropathy (Yang et al., 2019), and cisplatin-induced nephrotoxicity models (Yoshida et al., 2014). Here, we sought to investigate whether ML290 can prevent apoptosis and aberrant matrix remodeling in the unilateral ureteral obstruction (UUO) mouse model.

## Materials and Methods

### Animals

This study used homozygous humanized RXFP1 (*hRXFP1/hRXFP1*; Kaftanovskaya et al., 2019) and *Rxfp1*-deficient male mice (Kaftanovskaya et al., 2015) on C57BL/6 background previously generated in our laboratory. All mice were bred and housed on a 12:12 h day/night cycle at a room temperature of 20 ± 2°C in the Florida International University Animal Care Facility. UUO procedures were performed on 6 weeks old mice under isoflurane anesthesia. Briefly, the ureter was ligated at two different positions to separate the kidney and bladder; one near the renal pelvis and the other one close to the bladder. Un-operated right kidneys were used as contralateral controls in this study. A day after the UUO surgery, mice were randomly allocated to receive either vehicle [3% DMSO, 10% 1-Methyl-2-pyrrolidinone (NMP), 17% Kolliphor^®^ HS 15, 29% Poly (ethylene glycol) 400, and 41% phosphate-buffered saline] or ML290 (30 mg/kg; Wilson et al., 2018) *via* intraperitoneal injections for five consecutive days. Two days after the last injections, mice were anesthetized with isoflurane followed by cardiac puncture for blood collection. 20 μl of serum was used for osmolality measurement using the Advanced Micro Osmometer Model 3300 (Advanced Instruments, Norwood, MA). All experimental procedures were reviewed and approved by the FIU Institutional Animal Care and Use Committee under protocol AN16-003.

### Quantitative Real-Time PCR

Total RNA was isolated from the kidneys by column purification using a Direct-zol RNA MiniPrep Plus kit (Zymo Research, Irvine, CA) following manufacturer’s protocol. First strand complementary DNA (cDNA) was synthesized using the Verso cDNA synthesis kit (Thermo Scientific, Waltham, MA) and 1 μg RNA in a final reaction volume of 20 μL. Target gene expression was assessed by quantitative PCR using –2^ΔΔCt^ method with *Gapdh* as the housekeeping gene. Mouse or human (*RXFP1*)-specific PCR primers (Eurofins Scientific, Louisville, KY) were designed using the Universal Probe Library Web site (Roche, Indianapolis, IN; Table 1), and PCR reaction was determined by SYBR Green chemistry.

**Table 1:** Gene sequences of primers used for quantitative real-time PCR experiments.

Gene		Sequence 5' to 3'	
*Acta2*	Fwd	ACCACCCACCCAGAGTG	
	Rev	GTCTTCCTCTTCACACATAGC	
*Bax*	Fwd	GTGAGCGGCTGCTTGTCT	
	Rev	GGTCCCGAAGTAGGAGAGGA	
*Bcl2*	Fwd	GTACCTGAACCGGCATCTG	
	Rev	GGGGCCATATAGTTCCACAA	
*Cd68*	Fwd	TCCACTGTTGGCCCTCAC	
	Rev	CCCCTTGGACCTTGGACTA	
*Col1a1*	Fwd	CCTCAGGGTATTGCTGGACAAC	
	Rev	ACCACTTGATCCAGAAGGACCTT	
*Col3a1*	Fwd	TCCCCTGGAATCTGTGAATC	
	Rev	TGAGTCGAATTGGGGAGAAT	
*Col4a2*	Fwd	GACCGAGTGCGGTTCAAAG	
	Rev	CGCAGGGCACATCCAACTT	
*Gapdh*	Fwd	AACGACCCCTTCATTGAC	
	Rev	TCCACGACATACTCAGCAC	
*Mmp2*	Fwd	AACTTTGAGAAGGATGGCAAGT	
	Rev	TGCCACCCATGGTAAACAA	
*Pparg*	Fwd	GCTGTCATTATTCTCAGTGGAGAC	
	Rev	GAACAGCTGAGAGGACTCGG	
*RXFP1*	Fwd	TGACATCTGGTTCTGTCTTCTTCT	
	Rev	CAGTCGTCCACACCGTTACA	
*Tgfb1*	Fwd	TGGAGCAACATGTGGAACTC	
	Rev	CAGCAGCCGGTTACCAAG	
*Timp1*	Fwd	GCAAAGAGCTTTCTCAAAGACC	
	Rev	AGGGATAGATAAACAGGGAAACACT	
*Timp2*	Fwd	CGTTTTGCAATGCAGACGTA	
	Rev	GGAATCCACCTCCTTCTCG	
*Tnf*	Fwd	TTGTCTTAATAACGCTGATTTGGT	
	Rev	GGGAGCAGAGGTTCAGTGAT	
*Vim*	Fwd	CCAACCTTTTCTTCCCTGAA	
	Rev	TGAGTGGGTGTCAACCAGAG	

### Western Blot Analysis

Thirty μg of kidney protein lysates was subjected to SDS-PAGE and Western blot analysis using primary mouse/rabbit antibodies for phospho-p44/42 MAPK (pErk1/2; Thr202/Tyr204; 1:500, catalog no. 4370S, Cell Signaling, Danvers, MA), p44/42 MAPK (Erk1/2; 1:1,000, catalog no. 4695S, Cell Signaling), platelet-derived growth factor receptor-β (PDGFR-β; 1:300, catalog no. sc-374573, Santa Cruz Biotechnology, Dallas, TX), peroxisome proliferator-activated receptor gamma (PPARG; 1:250, catalog no. sc-7273, Santa Cruz Biotechnology), and alpha smooth muscle actin (αSMA; 1:500, catalog no. ab5694, Abcam, Cambridge, MA) overnight at 4°C. To normalize for the amount of protein, membranes were re-probed with a loading control antibody (β-tubulin, 1:1,000, catalog no. 05-661, Millipore Sigma, Burlington, MA). Protein expression was detected using the SuperSignal West Pico Chemiluminescent Substrate (catalog no. 34080, Thermo Scientific) after incubation with either anti-rabbit or anti-mouse horseradish peroxidase-conjugated secondary antibody (Promega, Madison, WI). Original images of Western blots are shown in Supplementary Figures 1–8.

### TUNEL Staining

Paraformaldehyde-fixed paraffin-embedded kidneys were used for *in situ* apoptosis detection using the ApopTag Plus Peroxidase kit (Millipore Sigma) following manufacturer’s protocol. Non-specific binding of enzyme-conjugate was evaluated in the absence of terminal deoxynucleotidyl transferase (TdT) labeling. At least 10 non-overlapping images were analyzed for each animal using the Carl Zeiss Axio A1 microscope with an AxioCam MRc5 CCD camera (Oberkochen, Germany) at 20× magnification, and TUNEL-positive cells were counted using a hand tally counter in a blinded manner.

### Sirius Red Staining

Paraformaldehyde-fixed paraffin-embedded kidneys were sectioned at 4.5 μm thickness and stained with 0.1% sirius red solution (Electron Microscopy Sciences, Hatfield, PA, United States; Ng et al., 2017). At least 10 non-overlapping bright field images at 20× magnification were analyzed for each animal using the Carl Zeiss Axio A1 microscope with an AxioCam MRc5 CCD camera (Oberkochen, Germany). Staining was quantified using ImageJ software (NIH, Bethesda, MD; Schneider et al., 2012) and presented as percentage of stained area within the analyzed image.

### Mouse Metalloproteinase Discovery Array

Fifty μl of undiluted kidney protein lysates was used for the detection of MMP2, MMP3, MMP8, MMP12, and proMMP9 by multiplexing technology (Eve Technologies, Calgary, AB). Protein concentration of each sample was calculated by fitting the fluorescence intensity values using a cubic spline regression applied against the standard curve fluorescence.

### Statistical Analysis

Two-way ANOVA with Tukey’s multiple comparison test assessed statistical differences in kidney weight, TUNEL staining, gene, and protein expression between groups. Un-paired student *t*-test assessed statistical differences in serum osmolality between vehicle- and ML290-treated mice. A level of *p* < 0.05 was considered statistically significant. All results are presented as mean ± SEM, where *n* represents the number of mice per group.

## Results

### Anti-apoptotic Actions of ML290

**Figure 1:**
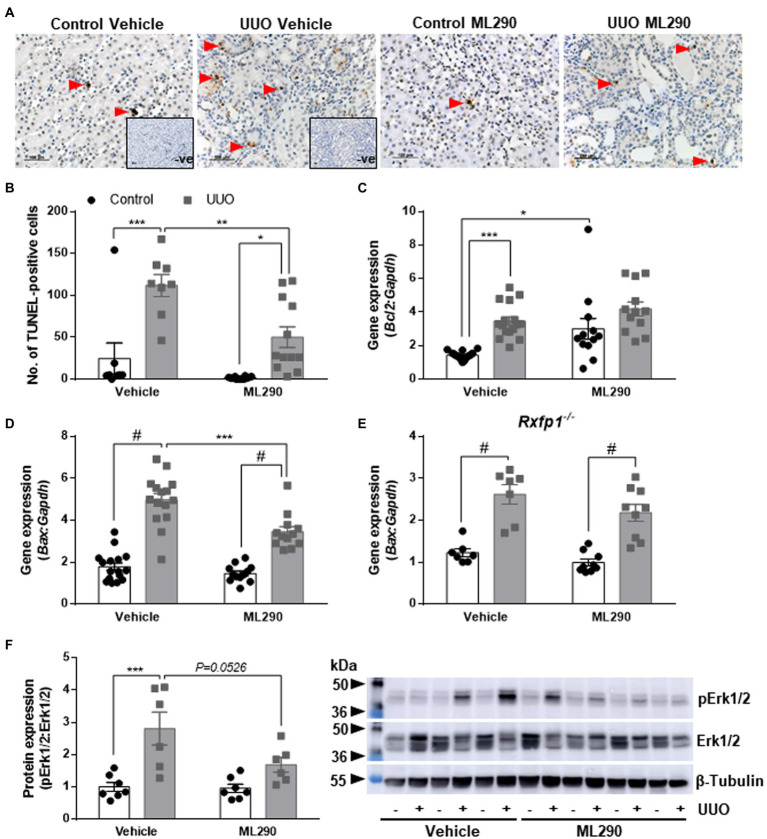
**(A)**: Representative images of TUNEL staining. Red arrows indicate specific staining for apoptotic cells. There was no positive brown staining in negative control (without TdT enzyme; *inset*). Scale bar = 100 μm. **(B)**: Quantitative analysis of TUNEL-positive cells in the control and unilateral ureteral obstruction (UUO) kidneys from vehicle- and ML290-treated humanized mice (*n* = 8–12 mice/group). **(C–E)**: *Bcl2* **(C)** and *Bax* gene expression in the control and UUO kidneys from vehicle- and ML290-treated humanized **(D)** (*n* = 12–16 mice/group) and *Rxfp1*-deficient **(E)** (*n* = 7–9 mice/group) mice. Gene expression is normalized to the housekeeping gene *Gapdh* and presented as fold change to control kidneys from vehicle-treated mice. **(F)**: Densitometric ratio of phospho-Erk1/2 (44, 42 kDa) to total Erk1/2 (44, 42 kDa) protein expression in the control and UUO kidneys from vehicle- and ML290-treated humanized mice (*n* = 6–7 mice/group). β-tubulin (55 kDa) was used as the loading control. ^*^p < 0.05, ^**^p < 0.01, ^***^p < 0.001, and ^#^*p* < 0.0001.

Unilateral ureteral obstruction induced a significant increase of apoptosis in the kidney from both vehicle- (*p* < 0.001) and ML290-treated (*p* = 0.01) mice (Figures 1A,B). Daily administration of ML290 decreased apoptosis in UUO kidney when compared to vehicle-treated mice, as evidenced by a significant (*p* = 0.004) reduction in the number of TUNEL-positive cells (Figures 1A,B). Interestingly, there was a significant (*p* < 0.001) increase in the expression of the pro-survival gene, *Bcl2* in the UUO kidney of vehicle-treated mice when compared to their contralateral controls (Figure 1C). This effect was abolished in ML290-treated mice, with a concomitant increase (*p* = 0.01) in *Bcl2* expression when compared to vehicle-treated mice in the contralateral control kidneys (Figure 1C). Consistent with our TUNEL staining, the pro-apoptotic gene, *Bax*, was significantly (*p* < 0.0001) upregulated in UUO kidneys from both vehicle- and ML290-treated mice when compared to their contralateral controls (Figure 1D). ML290 treatment significantly (*p* < 0.001) downregulated *Bax* expression in the UUO kidney when compared to vehicle-treated mice (Figure 1D). Importantly, *Rxfp1^−/−^* mice showed significant (*p* < 0.0001) increase in *Bax* expression after UUO surgery, but it was unaffected in the UUO kidneys of vehicle- and ML290-treated mice, indicating the specificity of the molecule in our humanized mice (Figure 1E). UUO surgery enhanced the pro-apoptotic activity of Erk1/2 in vehicle-treated mice, as evidenced by a significant (*p* < 0.001) increase in the ratio of phosphorylated Erk1/2 to total Erk1/2 protein (Figure 1F). These phenotypic changes induced by UUO were not apparent in the kidneys of ML290-treated mice (Figure 1F). There was a reduction (*p* = 0.05) in Erk1/2 activity in the UUO kidneys of ML290-treated mice when compared to the vehicle group, but it did not reach significance (Figure 1F). Despite the marked anti-apoptotic role of ML290 *in vivo*, the kidney weight of these mice was unaffected by either the UUO procedure or treatment after normalization to body weight. Daily intraperitoneal administration of ML290 did not affect serum osmolality (vehicle 388.1 ± 7.685 mOsm vs. ML290 381.3 ± 6.343 mOsm) in UUO mice when compared to vehicle-treated group.

### Effects of ML290 on Myofibroblast Differentiation

**Figure 2:**
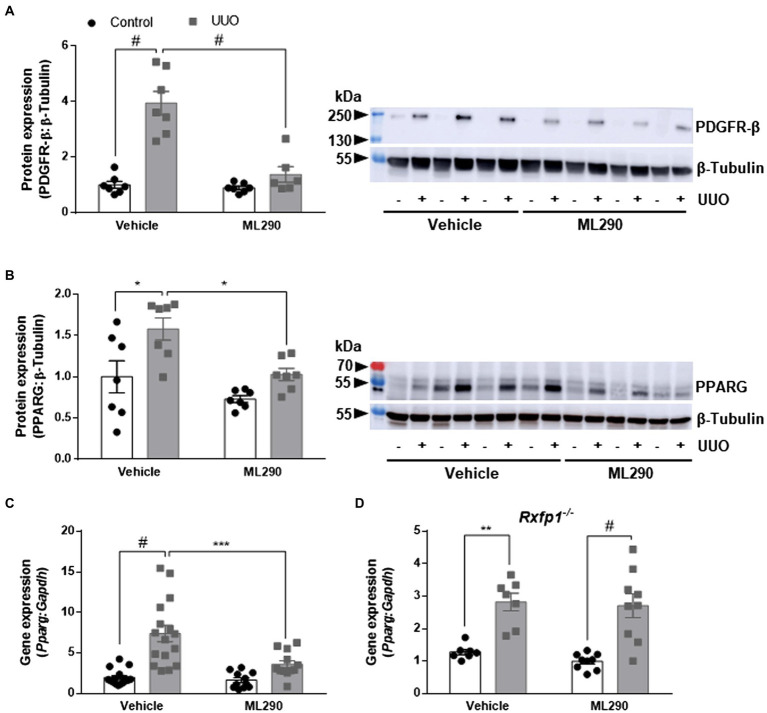
**(A,B)**: Densitometry of PDGFR-β **(A)** (180–190 kDa) and PPARG **(B)** (54 kDa) protein expression in the control and UUO kidneys from vehicle- and ML290-treated humanized mice (*n* = 6–7 mice/group). β-tubulin (55 kDa) was used as the loading control. **(C,D)**: *Pparg* gene expression in the control and UUO kidneys from vehicle- and ML290-treated humanized **(C)** (*n* = 12–16 mice/group) and *Rxfp1*-deficient **(D)** (*n* = 7–9 mice/group) mice. Gene expression is normalized to the housekeeping gene *Gapdh* and presented as fold change to control kidneys from vehicle-treated mice. ^*^p < 0.05, ^**^p < 0.01, ^***^p < 0.001, and ^#^*p* < 0.0001.

Unilateral ureteral obstruction significantly upregulated PDGFR-β (*p* < 0.0001; Figure 2A) and PPARG (*p* = 0.02; Figure 2B) protein expression in the kidneys of vehicle-treated mice when compared to their contralateral controls. This phenotype was abolished in ML290-treated mice, and there was a significant reduction in both the PDGFR-β (*p* < 0.0001; Figure 2A) and PPARG (*p* = 0.02; Figure 2B) expression in the UUO kidneys when compared to vehicle-treated mice. Similarly, *Pparg* gene expression was significantly (*p* < 0.0001) increased in the UUO kidneys of vehicle-treated, but not ML290-treated mice (Figure 2C). Consistent with PPARG protein expression in the UUO kidneys, ML290 significantly (*p* < 0.001) downregulated *Pparg* gene expression as well (Figure 2C). ML290-mediated actions on *Pparg* were not observed in the UUO kidneys of *Rxfp1^−/−^* mice, despite the significant increase of this nuclear transcription factor after UUO regardless of treatment (vehicle *p* = 0.002 and ML290 *p* < 0.0001; Figure 2D).

**Figure 3:**
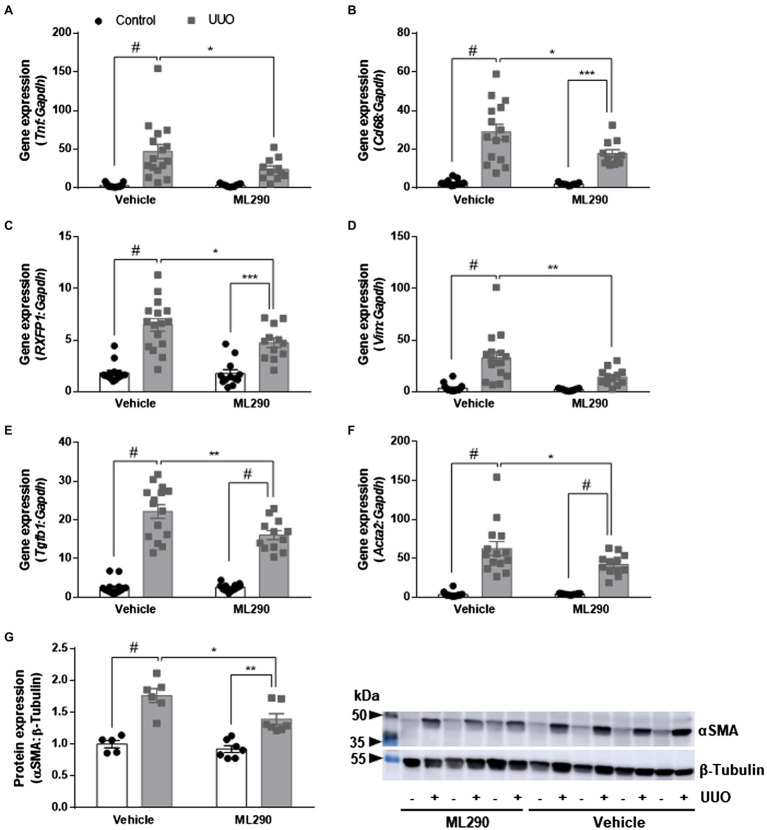
**(A*–*F)**: *Tnf* **(A)**, *Cd68* **(B)**, *RXFP1* **(C)**, *Vim* **(D)**, *Tgfb1* **(E)**, and *Acta2* **(F)** gene expression in the control and UUO kidneys from vehicle- and ML290-treated humanized mice (*n* = 12–16 mice/group). Gene expression is normalized to the housekeeping gene *Gapdh* and presented as fold change to control kidneys from vehicle-treated mice. **(G)**: Densitometry of αSMA (42 kDa) protein expression in the control and UUO kidneys from vehicle- and ML290-treated humanized mice (*n* = 5–7 mice/group). β-tubulin (55 kDa) was used as the loading control. ^*^p < 0.05, ^**^p < 0.01, and ^#^*p* < 0.0001.

UUO promoted inflammation in the kidneys of vehicle-treated but not ML290-treated mice, as evidenced by a significant (*p* < 0.0001) increase in *Tnf* gene expression when compared to their contralateral control kidney (Figure 3A). ML290 suppressed this inflammatory response by significantly (*p* = 0.04) reducing the expression of *Tnf* in the UUO kidney (Figure 3A). Inflammation in the kidney is often accompanied by macrophage infiltration. We demonstrated that UUO significantly increases the gene expression of a macrophage marker, *Cd68* in both vehicle- (*p* < 0.0001) and ML290-treated (*p* < 0.001) mice (Figure 3B). Consistent with the reduction of *Tnf* in the UUO kidneys of ML290-treated mice, *Cd68* expression was significantly (*p* = 0.01) downregulated when compared to vehicle-treated mice (Figure 3B).

Quantitative assessment of human *RXFP1* by qPCR indicated significant increase of the receptor in UUO kidney of both vehicle- (*p* < 0.0001) and ML290-treated (*p* < 0.001) mice (Figure 3C). *RXFP1* expression was significantly (*p* = 0.05) reduced in the UUO kidneys of ML290-treated mice compared to the vehicle group, an effect that may be associated with a reduction in fibroblasts activation (Figure 3C). Consistent with these findings, we found that UUO significantly (*p* < 0.0001) upregulated gene expression of several fibroblastic markers, such as *Vim*, *Tgfb1*, and *Acta2* in vehicle-treated mice when compared to their contralateral control kidneys (Figures 3D–F). Notably, ML290 treatment for 5 days significantly reduced the expression of these fibroblastic markers (*Vim p* = 0.003, *Tgfb1 p* = 0.002, and *Acta2 p* = 0.04) in the UUO kidneys when compared to vehicle-treated mice (Figures 3D–F). Despite the profound action of ML290 to suppress fibroblast activation in the UUO kidney, *Tgfb1* and *Acta2* expression remained significantly (*p* < 0.0001) elevated when compared to their contralateral controls (Figures 3E,F). Protein analysis of αSMA was consistent with the expression at transcriptional level, revealing a significant upregulation of this protein induced by UUO in both vehicle- (*p* < 0.0001) and ML290-treated (*p* = 0.001) mice (Figure 3G). Collectively, we showed that ML290 treatment effectively prevented myofibroblast differentiation in the UUO kidneys, with significant (*p* = 0.02) inhibition on the αSMA production (Figure 3G).

### Extracellular Matrix Remodeling Actions of ML290

**Figure 4:**
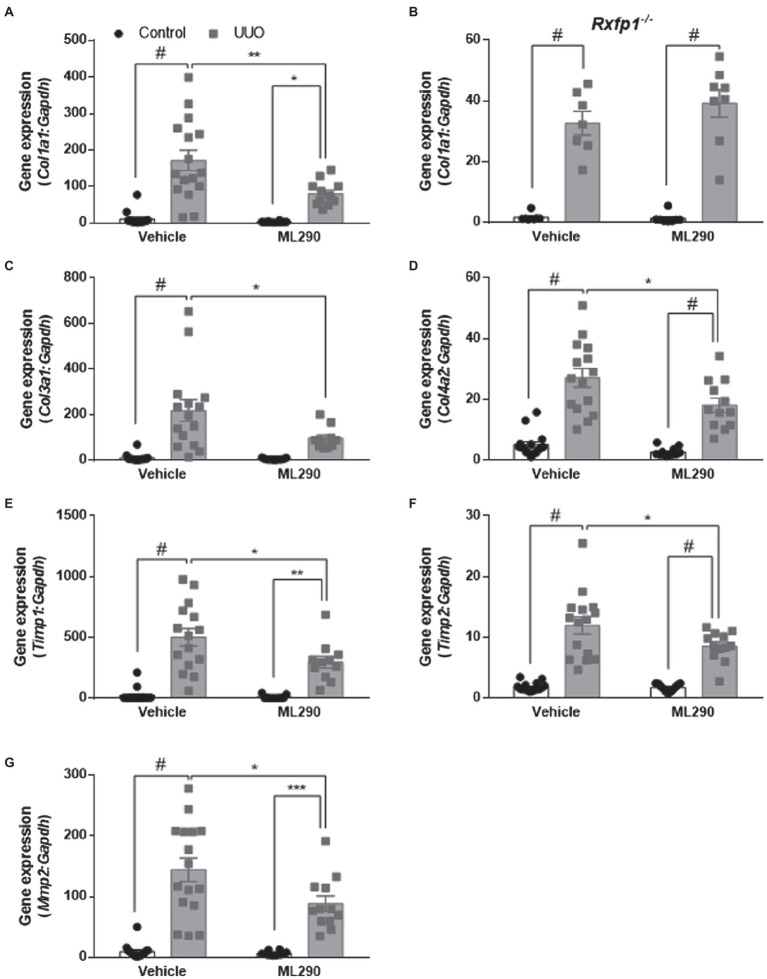
**(A,C*–*G)**: *Col1a1* **(A)**, *Col3a1* **(C)**, *Col4a2* **(D)**, *Timp1* **(E)**, *Timp2* **(F)**, and *Mmp2* **(G)** gene expression in the control and UUO kidneys from vehicle- and ML290-treated humanized mice (*n* = 12–16 mice/group). **(B)**: *Col1a1* gene expression in the control and UUO kidneys from vehicle- and ML290-treated *Rxfp1*-deficient mice (*n* = 7–8 mice/group). Gene expression is normalized to the housekeeping gene *Gapdh* and presented as fold change to control kidneys from vehicle-treated mice. ^*^p < 0.05, ^**^p < 0.01, ^***^p < 0.001, and ^#^*p* < 0.0001.

**Figure 5:**
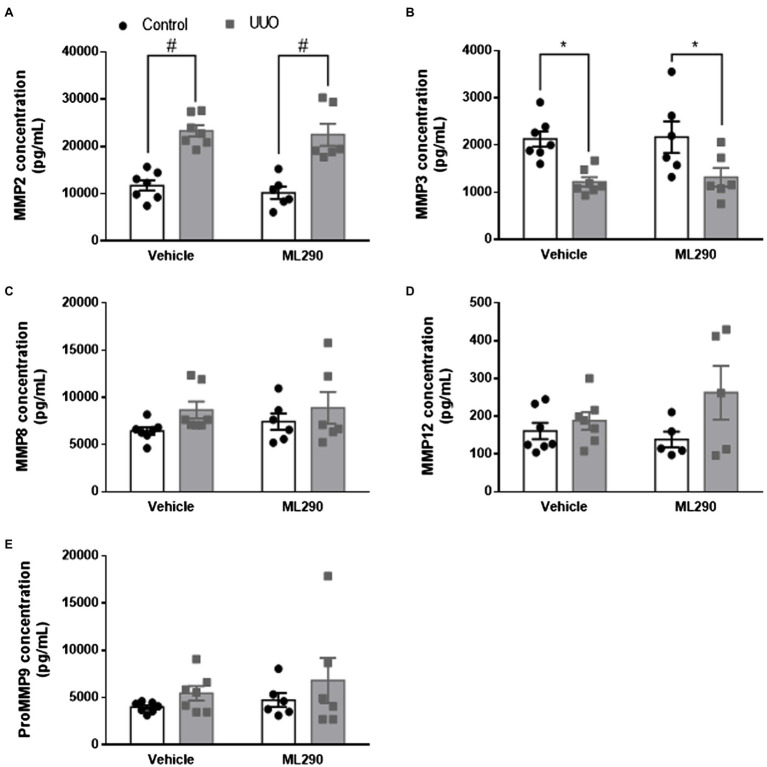
**(A*–*E)**: MMP2 **(A)**, MMP3 **(B)**, MMP8 **(C)**, MMP12 **(D)**, and proMMP9 **(E)** concentration in the control and UUO kidneys from vehicle- and ML290-treated humanized mice (*n =* 5–7 mice/group). Protein concentration is expressed as pg/ml. ^*^p < 0.05 and ^#^*p <* 0.0001.

Aberrant extracellular matrix accumulation was observed in the UUO kidneys of vehicle-treated mice when compared to their contralateral controls, as shown by a significant (*p* < 0.0001) increase in *Col1a1*, *Col3a1*, and *Col4a2* gene expression (Figures 4A,C,D). In the UUO kidneys of ML290-treated mice, *Col1a1* (*p* = 0.02) and *Col4a2* (*p* < 0.0001) expression remained significantly elevated when compared to their contralateral controls (Figures 4A,D). There was, however, a significant reduction in the expression of these genes (*Col1a1 p* = 0.002, *Col3a1 p* = 0.02, and *Col4a2 p* = 0.02) in the UUO kidneys of ML290-treated mice when compared to the vehicle group (Figures 4A,C,D), suggesting the matrix remodeling actions of ML290 *in vivo*. To confirm the ML290-mediated inhibition on fibrogenesis was specific on the human *RXFP1*, we showed that *Col1a1* expression was unaffected by ML290 treatment in the *Rxfp1^−/−^* mice, despite significant (*p* < 0.0001) increase of this gene in the UUO kidneys of vehicle- and ML290-treated mice (Figure 4B). Interestingly, we found that the interstitial collagen content was unaffected by ML290 treatment in the UUO kidneys when compared to vehicle-treated mice, as evidenced by similar level of sirius red staining in both the UUO groups (Supplementary Figure 9). In UUO kidneys, the aberrant accumulation of extracellular matrix was associated with a significant increase of *Timp1* and *Timp2* expression in the kidneys of both vehicle- (*p* < 0.0001) and ML290-treated (*Timp1 p* = 0.001 and *Timp2 p* < 0.0001) mice (Figures 4E,F). Interestingly, the extracellular matrix degrading gene, *Mmp2* was also significantly upregulated in the UUO kidneys of vehicle- (*p* < 0.0001) and ML290-treated (*p* < 0.001) mice (Figure 4G). These findings suggest that the concomitant increase in *Mmp2* may be a compensatory mechanism to overcome the impaired matrix turnover induced by UUO. In comparison to the UUO kidneys from vehicle-treated mice, ML290-treated mice had significantly lower *Timp1* (*p* = 0.02), *Timp2* (*p* = 0.04), and *Mmp2* (*p* = 0.02) expression (Figures 4E–G), an effect that may be due to the already reduced collagen in their UUO kidneys. Despite the ability of ML290 to reduce *Mmp2* expression at the transcriptional level, MMP2 protein expression was not significantly different from UUO kidneys of vehicle-treated mice (Figure 5A). UUO significantly (*p <* 0.0001) upregulated MMP2 level and significantly (*p <* 0.05) downregulated MMP3 level in the kidneys of both vehicle- and ML290-treated mice (Figures 5A,B). MMP8 (Figure 5C), MMP12 (Figure 5D), and proMMP9 (Figure 5E) levels were not affected by either UUO or ML290 treatment.

## Discussion

The aims of this study were to investigate the therapeutic potential of ML290 to reduce apoptosis in the kidney and to test the hypothesis that the non-peptide small molecule RXFP1 agonist ML290 suppresses aberrant extracellular matrix remodeling in the UUO mice. We demonstrated that daily administration of ML290 has potent anti-apoptotic actions in the UUO kidneys, primarily through the suppression of pro-apoptotic markers and promotion of pro-survival activity. Furthermore, ML290 impedes pericyte-myofibroblast differentiation in the UUO kidneys and inhibits fibrogenesis. The ML290-mediated effects were at least in part attributed to a reduction in inflammation and macrophage infiltration in the UUO kidneys.

Very little work to date has assessed the anti-apoptotic effects of relaxin in the kidneys. The majority of the research has focused on investigating relaxin’s anti-apoptotic actions in cardiomyocytes (Moore et al., 2007; Ng et al., 2017) and hepatocytes (Kageyama et al., 2018; Lee et al., 2019). Here, we showed that activation of the human RXFP1 by ML290 in humanized mice potently prevented UUO-induced apoptosis in the kidneys. These effects were evident by the detection of DNA breakage that occurs during apoptosis, and subsequent changes in the pro-apoptotic gene, *Bax* expression at transcriptional level. Conventionally, activation of Erk1/2 is related to cell survival, but several studies suggest that Erk1/2 could promote cell death under certain stimuli (Pearson et al., 2001). It is possible that the balance between the activation of pro-apoptotic or pro-survival pathways underscores the signals transmitted by Erk1/2 and determines whether a cell undergoes apoptosis. In fact, we found that the pro-survival gene, *Bcl2* was simultaneously upregulated in the UUO kidneys, indicating a compensatory mechanism to overcome cell deaths that occurred in the UUO kidneys. Interestingly, *Bcl2* expression was significantly increased in the contralateral control kidneys of ML290-treated mice when compared to their vehicle-treated counterparts. These data may suggest that ML290 promotes cell survival in the healthy kidneys to compensate for the reduced function of the left kidneys (UUO), since there was no apparent reduction in *Bcl2* expression in the UUO kidneys of ML290-treated mice.

Furthermore, we demonstrated that UUO markedly upregulated expression of the receptor for PDGF-β only in vehicle-treated mice. This is consistent with our *Bcl2* data as PDGF-β is one of the multiple major survival factors in several cell types of mesenchymal origin (Eitner et al., 2002). The PDGF-β receptor can be activated by various mediators, including TGF-β, fibroblast growth factor, and PDGF itself (Haberstroh et al., 1993; Floege and Johnson, 1995). Indeed, we found that *Tgfb1* expression was increased in the UUO kidneys regardless of treatment, when there was significant activation of the PDGF-β receptor in these diseased kidneys. In line with these observations, we showed that PPAR-γ expression was significantly upregulated in the UUO kidneys of vehicle-treated mice, suggesting the enhanced differentiation of pericytes to myofibroblasts that ultimately contributes to the accumulation of pathological extracellular matrix in these kidneys. In addition to the aberrant remodeling of the extracellular matrix, overproduction of myofibroblasts in the kidneys also leads to inflammation and macrophage infiltration. These phenotypes were apparent in the UUO kidneys of vehicle-treated, but not ML290-treated mice. Notably, ML290 potently downregulated α-SMA and vimentin expression in the UUO kidneys to inhibit fibrogenesis in these diseased kidneys. Additionally, we found that human *RXFP1* expression was increased in the UUO kidneys of both vehicle- and ML290-treated mice, an effect that may be in part due to fibroblasts activation (Fallowfield et al., 2014). Consistent with the α-SMA and vimentin data, *hRXFP1* expression was reduced by ML290 treatment. Taken together, we demonstrated the target-specific actions of ML290 to regulate fibroblasts and pericyte-myofibroblast transition in this mouse model.

Relaxin is known to be a potent regulator of collagen turnover in the UUO mouse model (Hewitson et al., 2010; Huuskes et al., 2015). Here, we sought to determine whether the biased allosteric agonist of RXFP1 possesses similar action to the native ligand in this mouse model. ML290 treatment profoundly inhibited UUO-induced upregulation of collagen expression in the kidneys, an effect that may be attributed to its action on the tissue inhibitors of metalloproteinases (*TIMP1* and *TIMP2*). We found that TIMPs expression was increased in the UUO kidneys to inhibit the degradation of extracellular matrix. Although expression of TIMPs remained significantly higher than the contralateral control kidneys in ML290-treated mice, these metalloproteinases were significantly downregulated in the diseased kidneys when compared to vehicle-treated mice. Interestingly, we found that *MMP2* expression was reduced in the UUO kidneys after ML290 treatment, despite that it has been shown to activate MMP2 in the human cardiac fibroblasts (Kocan et al., 2017). It is possible that in the UUO kidneys where there was overproduction of collagen, MMP2 is activated to accelerate the degradation of matrix as a compensatory mechanism in these mice. Since there was lower level of collagen mRNA in the UUO kidneys of ML290-treated mice, activation of MMP2 was less pronounced. Surprisingly, we did not observe significant reduction in interstitial collagen content in the UUO kidneys of ML290-treated mice, despite the marked decrease in gene expression of several collagen markers (*Col1a1*, *Col3a1*, and *Col4a2*). This may be due to the short duration of our treatment in these UUO mice that was insufficient to induce morphological changes and collagen turnover in their kidneys.

Finally, it was important to demonstrate that the effects of ML290 resulted from activation of the human RXFP1, as this compound does not activate the rodent receptor (Huang et al., 2015; Ng et al., 2019a). We tested the gene expression of several markers that were markedly reduced by ML290 treatment, in the *Rxfp1^−/−^* mice. These genes were upregulated in the UUO kidneys regardless of vehicle or ML290 treatment, indicating that data obtained from the humanized mice resulted from activation of the human receptor.

## Conclusion

Our proof-of-concept study revealed for the first time that ML290 prevented UUO-induced apoptosis and extracellular matrix remodeling in the kidneys of humanized mice. Detailed mechanistic studies should be carried out in the future to delineate the specific pathways activated by ML290 in more physiologically relevant pre-clinical animal models. Although ML290 is safe and tolerable in mice (Wilson et al., 2018; Kaftanovskaya et al., 2019), it is also important to evaluate the hemodynamics and functional outcomes of ML290 treatment *in vivo*, in particular those from diseased pre-clinical animal models. Future studies on the therapeutic efficacies of ML290 now warrant such enhanced translational value for future drug development.

## Data Availability Statement

The raw data supporting the conclusions of this article will be made available by the authors, without undue reservation.

## Ethics Statement

The animal study was reviewed and approved by the Florida International University Institutional Animal Care and Use Committee under protocol AN16-003.

## Author Contributions

HN wrote the manuscript. HN, MS, BR, KW, JM, and AA designed the work or contributed to the acquisition, analysis, or interpretation of data for the work, critically revised the work for important intellectual content, agreed to be accountable for all aspects, including the accuracy or integrity of the work, and approved the final version of the work to be published. All authors contributed to the article and approved the submitted version.

### Conflict of Interest

The authors declare that the research was conducted in the absence of any commercial or financial relationships that could be construed as a potential conflict of interest.
