# Supplementary material for: Anti-apoptotic and Matrix Remodeling Actions of a Small Molecule Agonist of the Human Relaxin Receptor, ML290 in Mice With Unilateral Ureteral Obstruction
Source: Front Physiol. 2021 Jul 7;12:650769. doi: 10.3389/fphys.2021.650769 (PMC8293094; doi:10.3389/fphys.2021.650769)
Supplement: Supplementary file 1 [file Data_Sheet_1.PDF]

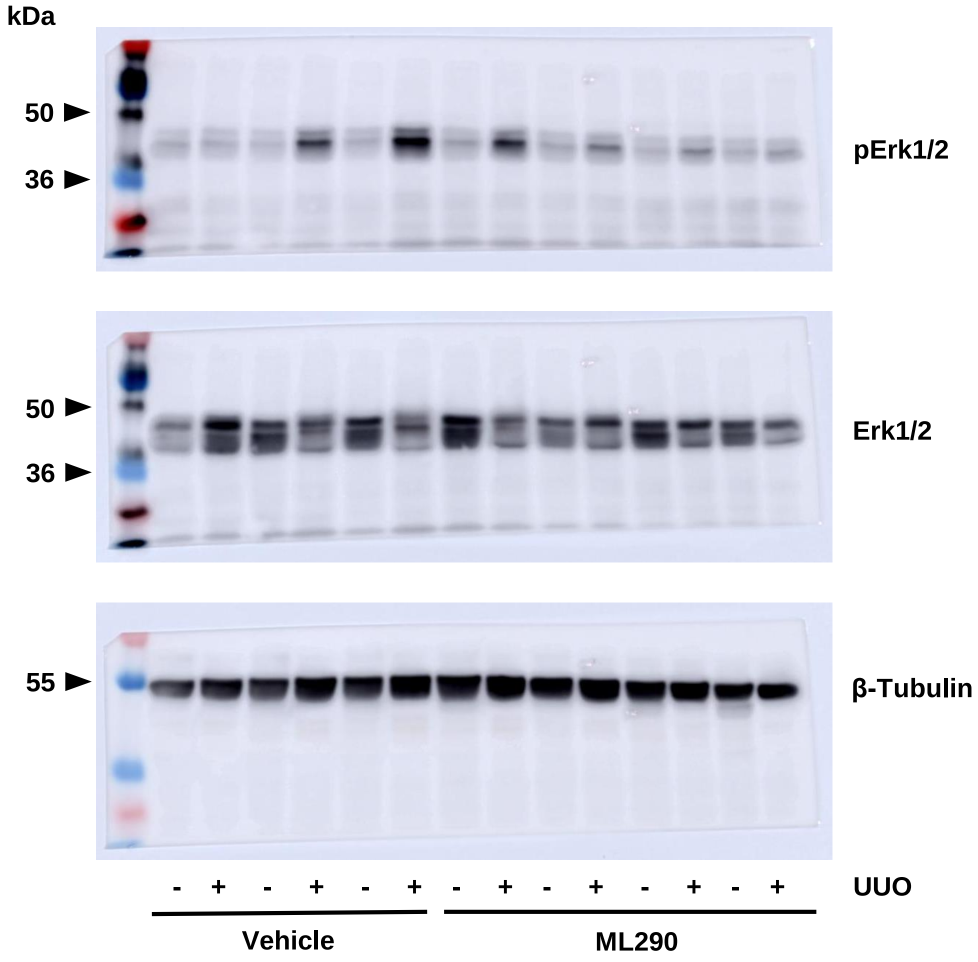


**Supplementary Figure 1**. Original western blot images of figure 1 (*F*). First blot of phospho-Erk1/2 (44, 42 kDa), total Erk1/2 (44, 42 kDa) and β-tubulin (55 kDa) from the control and UUO kidneys of vehicle- and ML290-treated humanized mice.

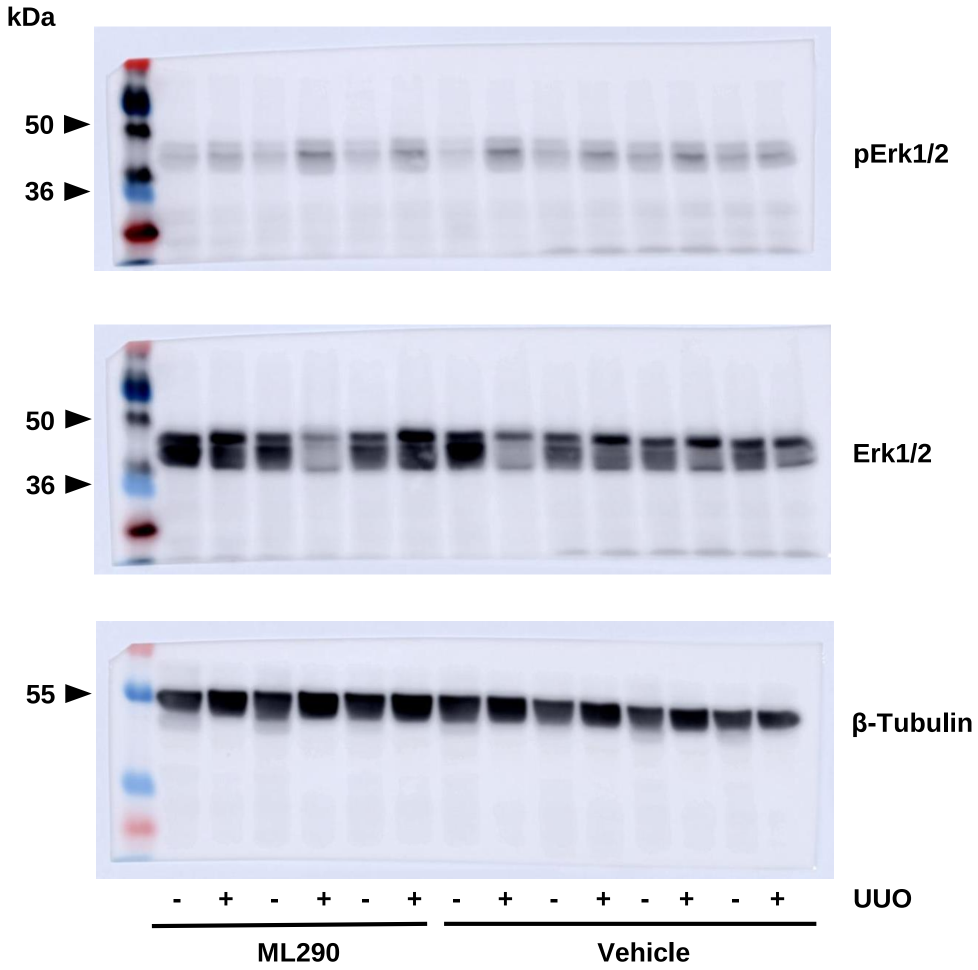


**Supplementary Figure 2**. Original western blot images of figure 1 (*F*). Second blot of phospho-Erk1/2 (44, 42 kDa), total Erk1/2 (44, 42 kDa) and β-tubulin (55 kDa) from the control and UUO kidneys of vehicle- and ML290-treated humanized mice.

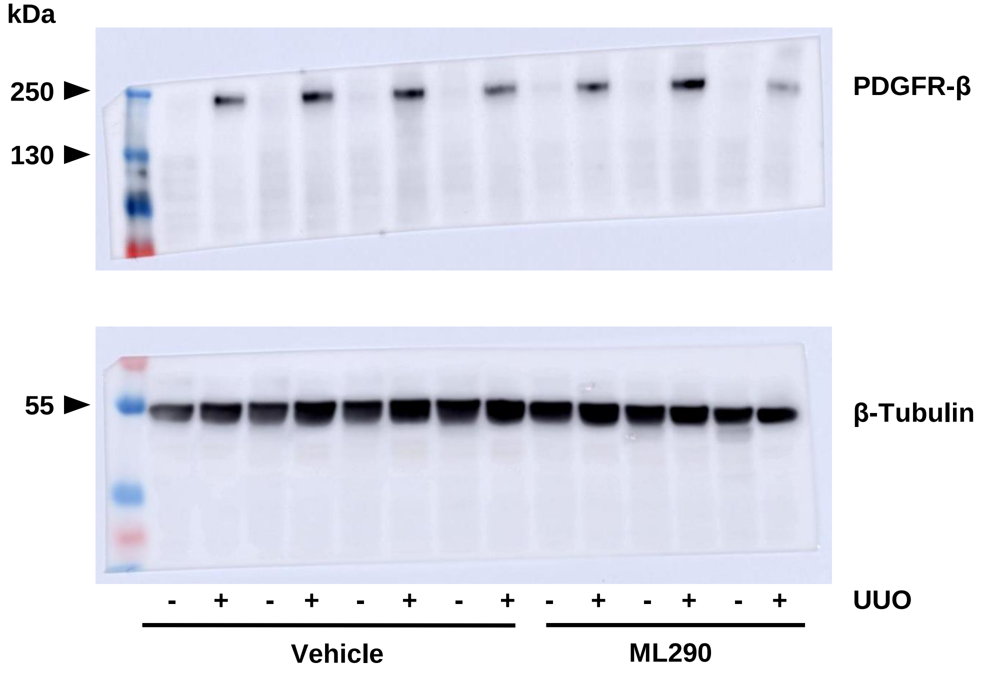


**Supplementary Figure 3**. Original western blot images of figure 2 (*A*). First blot of PDGFR-β (180-190 kDa) and β-tubulin (55 kDa) from the control and UUO kidneys of vehicle- and ML290-treated humanized mice. PDGFR-β was performed on the same gel as phospho-Erk1/2 (44, 42 kDa) and total Erk1/2 (44, 42 kDa) shown in supplementary figure 1.

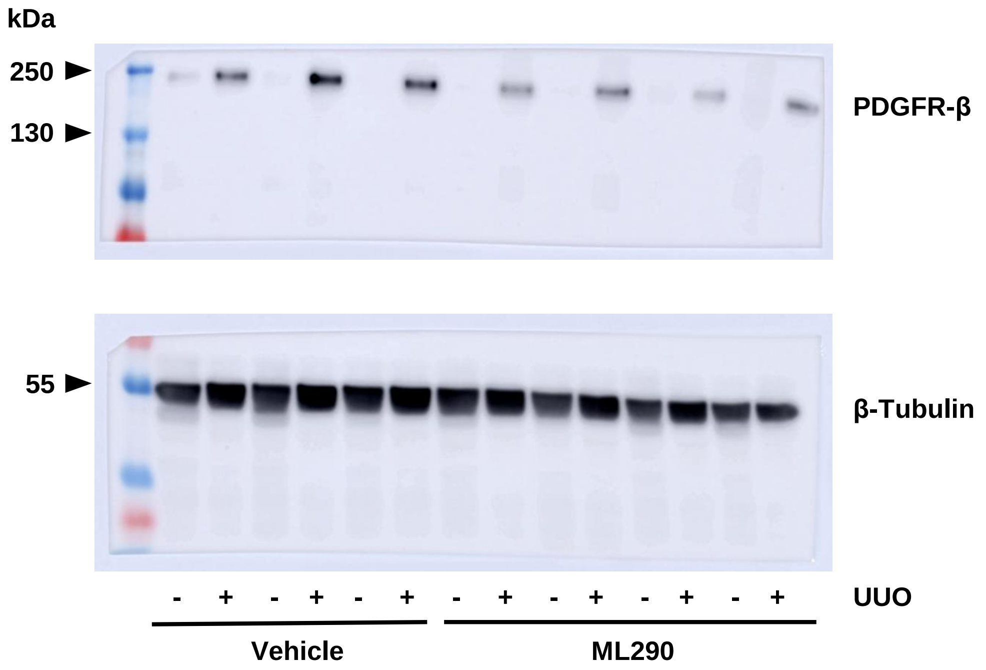


**Supplementary Figure 4**. Original western blot images of figure 2 (*A*). Second blot of PDGFR-β (180-190 kDa) and β-tubulin (55 kDa) from the control and UUO kidneys of vehicle- and ML290-treated humanized mice. PDGFR-β was performed on the same gel as phospho-Erk1/2 (44, 42 kDa) and total Erk1/2 (44, 42 kDa) shown in supplementary figure 2.

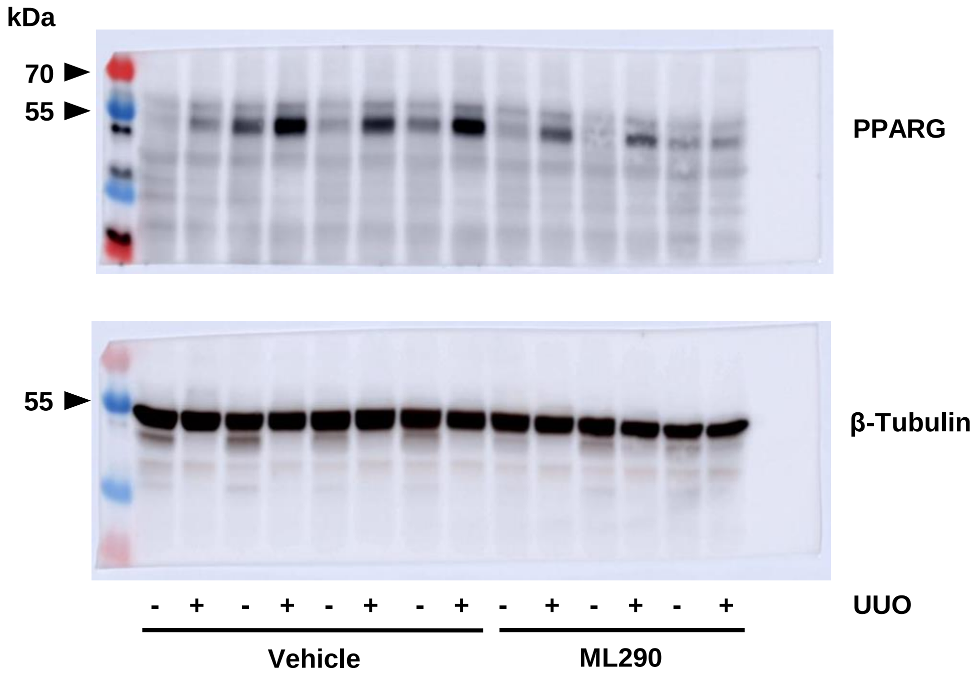


**Supplementary Figure 5**. Original western blot images of figure 2 (*B*). First blot of PPARG (54 kDa) and β-tubulin (55 kDa) from the control and UUO kidneys of vehicle- and ML290-treated humanized mice.

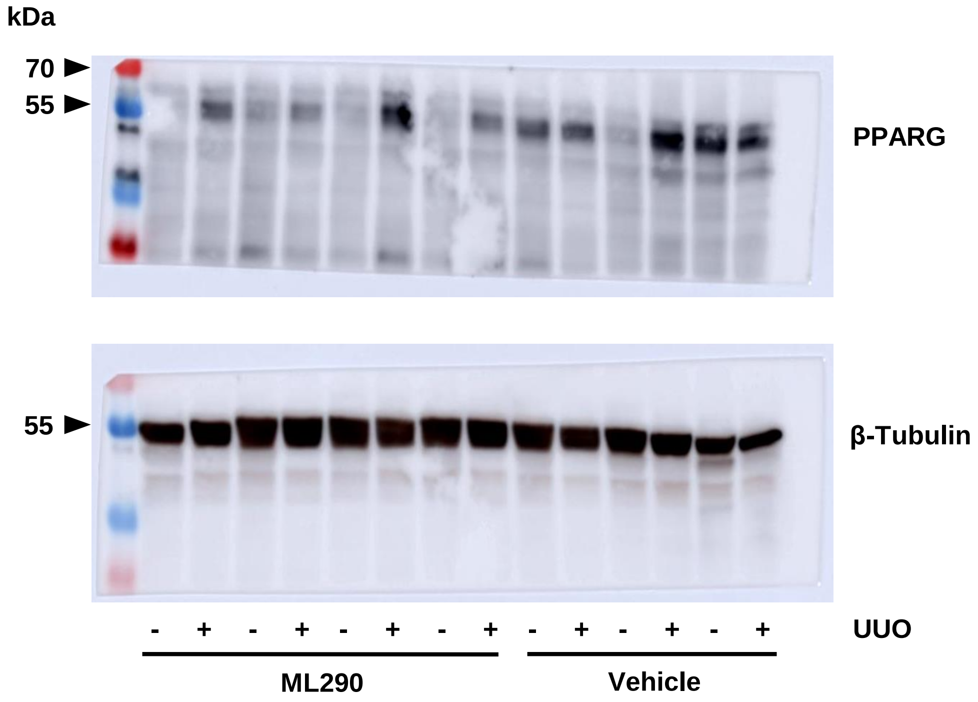


**Supplementary Figure 6**. Original western blot images of figure 2 (*B*). Second blot of PPARG (54 kDa) and β-tubulin (55 kDa) from the control and UUO kidneys of vehicle- and ML290-treated humanized mice.

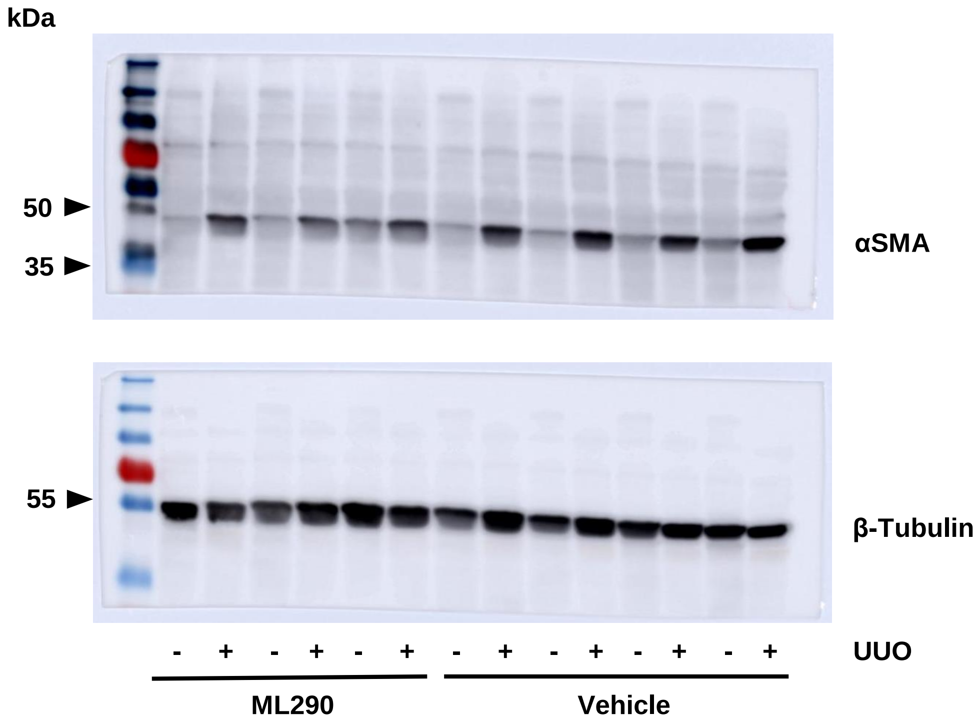


**Supplementary Figure 7**. Original western blot images of figure 3 (*G*). First blot of αSMA (42 kDa) and β-tubulin (55 kDa) from the control and UUO kidneys of vehicle- and ML290-treated humanized mice.

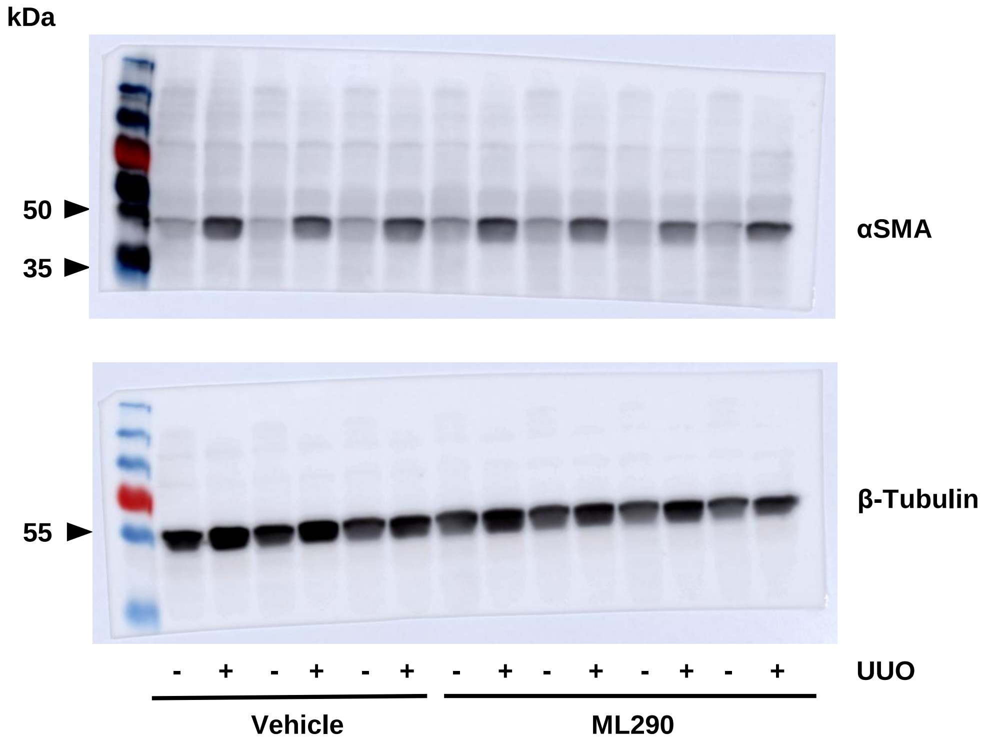


**Supplementary Figure 8**. Original western blot images of figure 3 (*G*). Second blot of αSMA (42 kDa) and β-tubulin (55 kDa) from the control and UUO kidneys of vehicle- and ML290-treated humanized mice.

(A)

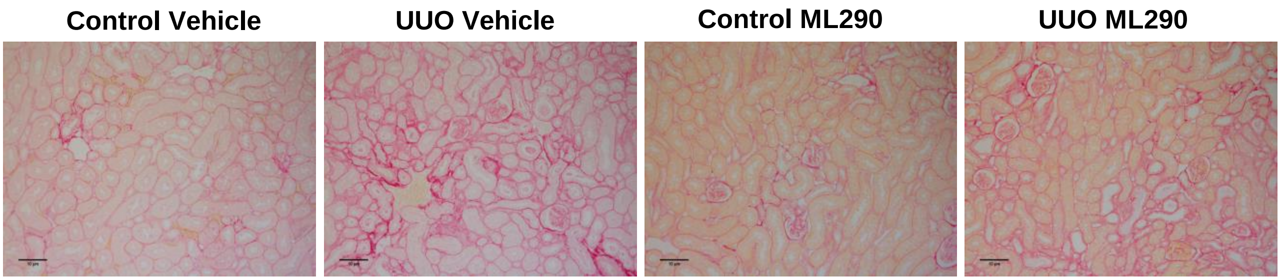


(B)

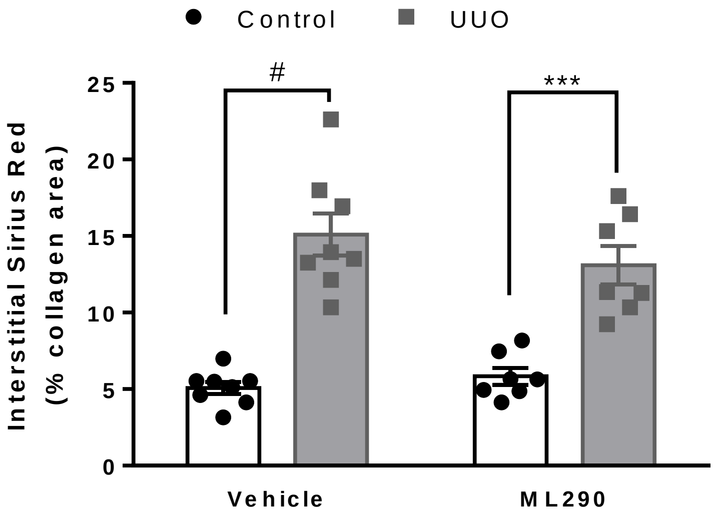


**Supplementary Figure 9**. *A*: Representative images of Sirius Red staining. Scale bar = 10 μm. *B*: Quantitative analysis of the percent positive Sirius Red area in the control and UUO kidneys from vehicle- and ML290-treated humanized mice ($n$ = 7-8 mice/group). ***$p < 0.001$, #$p < 0.0001$.
